# RNA-eXpress annotates novel transcript features in RNA-seq data

**DOI:** 10.1093/bioinformatics/btt034

**Published:** 2013-02-08

**Authors:** Samuel C. Forster, Alexander M. Finkel, Jodee A. Gould, Paul J. Hertzog

**Affiliations:** Centre for Innate Immunity and Infectious Diseases, Monash Institute of Medical Research, Monash University, Clayton, Victoria, Australia, 3168

## Abstract

**Summary:** Next-generation sequencing is rapidly becoming the approach of choice for transcriptional analysis experiments. Substantial advances have been achieved in computational approaches to support these technologies. These approaches typically rely on existing transcript annotations, introducing a bias towards known genes, require specific experimental design and computational resources, or focus only on identification of splice variants (ignoring other biologically relevant transcribed features contained within the data that may be important for downstream analysis). Biologically relevant transcribed features also include large and small non-coding RNA, new transcription start sites, alternative promoters, RNA editing and processing of coding transcripts. Also, many existing solutions lack accessible interfaces required for wide scale adoption. We present a user-friendly, rapid and computation-efficient feature annotation framework (RNA-eXpress) that enables identification of transcripts and other genomic and transcriptional features independently of current annotations. RNA-eXpress accepts mapped reads in the standard binary alignment (BAM) format and produces a study-specific feature annotation in GTF format, comparison statistics, sequence extraction and feature counts. The framework is designed to be easily accessible while allowing advanced users to integrate new feature-identification algorithms through simple class extension, thus facilitating expansion to novel feature types or identification of study-specific feature types.

**Availability and implementation:** RNA-eXpress software, source code, user manuals, supporting tutorials, developer guides and example data are available at http://www.rnaexpress.org.

**Contact:** paul.hertzog@monash.edu

**Supplementary information:** Supplementary data are available at *Bioinformatics* online.

## 1 INTRODUCTION

Recent advances in Next-Generation Sequencing (NGS) technology have revolutionized many aspects of biology. Applications range from genomic and metagenomic projects through to wide scale condition-specific whole transcriptome sequence (Wang *et al.*, 2009). Within the field of whole transcriptome sequencing, the primary benefit over traditional microarray is the capacity to read all expressed transcripts, yielding unbiased highly sensitive measurements, without the dependence on prior knowledge to specify probes. This capacity facilitates the discovery and quantification of novel genes, splice variants and non-coding RNAs in addition to known features.

The present challenge lies in extracting all biologically meaningful information from the vast amounts of data produced by NGS in a time- and resource-efficient manner. The assignment of biological significance relies on identification of transcriptional features, their analysis and quantitation of induced changes in expression. Many NGS studies reveal expressed features that are not annotated in the reference genomes, either because they are novel genes, transcripts or splice variants, or because they are composed of reads from precursor transcripts or intronic regions. Whole transcriptome analysis initially relies upon either prior knowledge of the genome for direct mapping or computationally and sequencing intensive *de-novo* transcript assembly (using tools such as TranABySS and Oases). Although comprehensive, these transcript reconstruction techniques are dependent on specific experimental designs, thus limiting application. Current genome-guided techniques for feature identification range from considering only gene annotation databases through to transcript assembly based on novel gene and splice variant identification directly from read information (such as performed with Scripture and Cufflinks) (Guttman *et al.*, 2010; Trapnell *et al.*, 2012). Although these features may identify novel splice variants, other transcribed features such as upstream transcribed regions, introns, downstream UTR regions and unprocessed RNA may not be considered. Although understanding the transcriptional response of protein-coding genes is critical, identifying and understanding the additional transcribed features can also provide significant insights into the regulatory controls and networks induced in the condition of study. Although present in many NGS datasets, this information is not widely analysed, thus excluding potentially important biologically relevant information.

In this light, we developed RNA-eXpress, a user-friendly computationally efficient analysis approach to perform flexible feature annotation, comparison, sequence extraction and read counting using NGS data from any experimental condition.

## 2 APPROACH

Algorithms have been implemented within the RNA-eXpress framework to enable efficient identification of splice variants, transcription start sites, UTRs, introns and non-coding RNA features from BAM-formatted next-generation sequencing data.

**Fig. 1.**
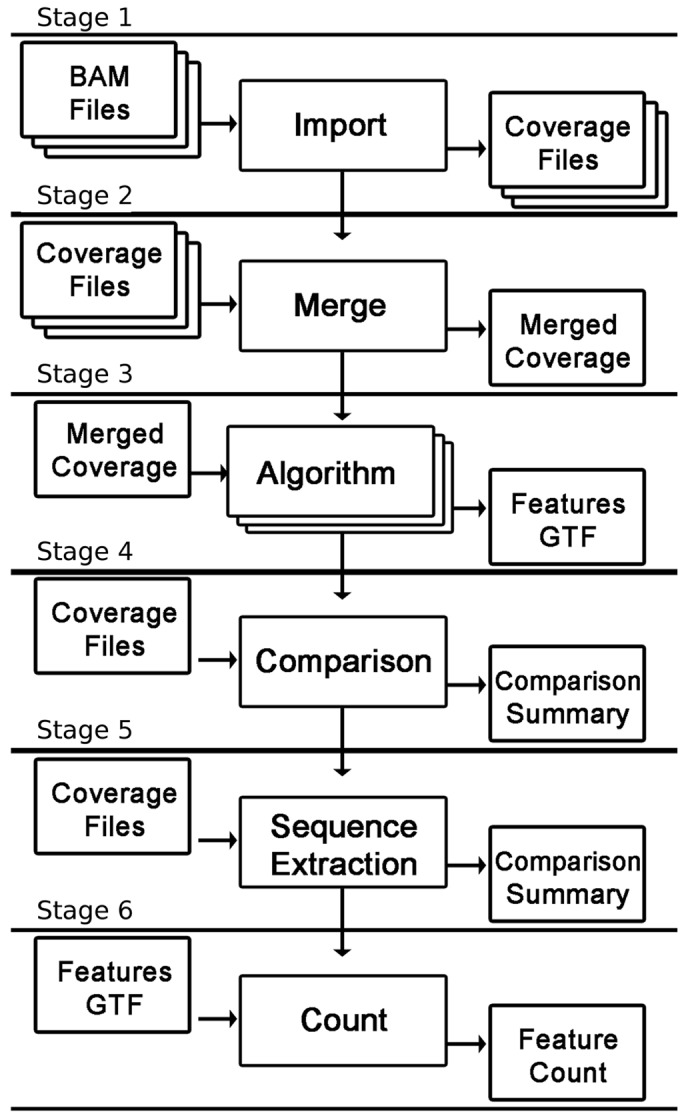
Diagrammatic representation of the RNA-eXpress workflow including input and output data production at each stage

The analysis process used by RNA-eXpress involves six phases: *data import and conversion*, *sample merging*, *algorithm execution*, and optionally *external GTF comparison, sequence extraction* and *read counting*. Comprehensive description of these stages, parameters required and output files are available in the user manual. Results at each stage are produced as independent output files, in standard formats providing flexibility to commence, complete and repeat analysis at any phase of the pipeline (Fig. 1).

The RNA-eXpress workflow is designed to accept input data in the standard BAM file format (Stage 1). Alternatively read-independent coverage information may also be imported in the WIG or BED formats. Regardless of input format, any number of samples or experimental conditions may be processed simultaneously. When identifying features, coverage is taken additively across samples from the same genome and merged to yield a maximal set of features (Stage 2). This merged coverage data structure represents the input for transcribed feature identification in the algorithm execution stage (Stage 3).

The algorithm class responsible for identifying features is designed to be easily extendable to implement additional feature identification algorithms while leveraging the GUI, threading and file workflow management capabilities provided by RNA-eXpress. Developers are encouraged to submit verified algorithms to the public algorithm repository. Multiple feature identification algorithms are already implemented with detailed descriptions of their intended usage available in the user manual. Briefly, the Transcripts algorithm provides comprehensive transcript identification even in complex mammalian genomes, the TSS algorithm predicts transcription start sites and UTR algorithm specifically identifies expressed 3′-UTRs to facilitate downstream miRNA-binding site prediction analysis.

To enable users to incorporate existing annotation information, an optional GTF merging stage involves comparison of identified features to an externally provided GTF file (Stage 4). At the completion of this stage, RNA-eXpress provides summary statistics, histograms and pie charts to facilitate comparison assessments and novel feature quantification. As final, optional stages, sequence extraction and read counting (Stages 5 and 6), using the generated GTF, can be performed for each sample providing inputs for downstream analysis.

## 3 IMPLEMENTATION

The RNA-eXpress algorithms are implemented in fully object-oriented Java using the Samtools Java library (Li *et al.*, 2009). The feature identification phase is implemented using the Java threading model to take full advantage of the available processors.

Comprehensive testing has yielded excellent results on both desktop computers and 32 core 100 GB RAM cluster nodes. A whole transcriptome experiment of nine samples of ∼500 million reads each can be analysed on the desktop machine in <5 h. This includes feature comparison and merging with the complete Ensembl database followed by exon sequence extraction and feature counting. This time can be reduced to ∼3 h in the cluster environment. This transcript algorithm compares favourably with existing Cufflinks and Scripture algorithms with respect to memory usage (2 Gb cf. to 6 Gb) and feature identification (Supplementary Fig. S1 and Table S1), with a number of novel features validated using RT-PCR (Supplementary Fig. 2). In addition RNA-eXpress provides significant advantages in run duration (5–10× faster), is capable of running on a desktop machine and provides greater flexibility and functionality than the existing solutions (Supplementary Fig. S1 and Table S1).

RNA-eXpress is freely available open source software with code, JAVA documentation and updates available. Example datasets with accompanying analysis tutorials are also available for download from the website. The authors welcome and encourage contributions to this open source project.

## 4 CONCLUSIONS

RNA-eXpress provides a method for extraction of the full detail contained within whole transcriptome next-generation sequencing experiments, thus facilitating comprehensive hypothesis-free analysis and knowledge extraction from these extensive datasets. The combination of a user-friendly GUI and low computational requirements makes RNA-eXpress accessible to the majority of users. The Java-based implementation ensures cross platform support and simple installation. With the growing availability of NGS datasets in novel conditions and species, RNA-eXpress provides a computational solution that is unbiased, usable and extensible.

## Supplementary Material

Supplementary Data
