# Supplementary material for: RNA-eXpress annotates novel transcript features in RNA-seq data
Source: Bioinformatics. 2013 Feb 8;29(6):810–2. doi: 10.1093/bioinformatics/btt034 (PMC3597146; doi:10.1093/bioinformatics/btt034)
Supplement: Supplementary Data [file supp_29_6_810__index.html]

RNA-eXpress annotates novel transcript features in RNA-seq data — RNA-eXpress annotates novel transcript features in RNA-seq data — RNA-eXpress annotates novel transcript features in RNA-seq data — Supplementary Data 

# RNA-eXpress annotates novel transcript features in RNA-seq data

## Supplementary Data

files

**Files in this Data Supplement:**

- Supplementary Data - doc file
