# Supplementary material for: RNA-eXpress annotates novel transcript features in RNA-seq data
Source: Bioinformatics. 2013 Feb 8;29(6):810–2. doi: 10.1093/bioinformatics/btt034 (PMC3597146; doi:10.1093/bioinformatics/btt034)
Supplement: Supplementary Data [file supp_btt034_Forster_etal_Supplementary.doc]

|  | **RNA-eXpress**  (1.2) | **Cufflinks**  (2.0.2) | **Scripture**  (beta2) |
| --- | --- | --- | --- |
| **Program Features** | | | |
| **Implementation Language** | Java | C++ | Java |
| **Operating System Support** | OS Independent  (Java 1.6) | Linux or Mac | OS Independent  (Java 1.6) |
| **Graphical User Interface** | Yes | No | No |
| **Optimal Operating Environment** | Desktop or Cluster | Cluster | Desktop or Cluster |
| **Support for Additional Algorithms** | Yes | No | No |
| **Designed to Detect Non-Transcript Features** | Yes | No | No |
| **Feature Quantification** | Yes | Yes | Yes |
| **Comparison To Known Features** | Yes | Yes | No |
| **Strand Specificity** | Yes | Yes | No |
| **Sequence Extraction** | Yes | No | No |
| **Automated Multiple Sample Analysis** | Yes | No | No |
| **Performance** | | | |
| **Region Runtime** | 0.6 s | 5.7 s | 1.2 s |
| **Chromosome Runtime** | 3.2 min | 41.9 min | 1.4 min |
| **Genome Runtime** | 45 min | 11.7 h | N/A |
| **Test Data Transcript Accuracy (Manual Curation)** | 96% | 94% | 91% |
| **Test Data Transcription Start Site Accuracy (Manual Curation)** | 97% | N/A | N/A |

**Supplementary Table 1: Comparison of the functionality and performance of RNA-eXpress with existing Cufflinks and Scripture solutions.** Features of RNA-eXpress include desktop support, flexible algorithm extension and the ability to detect features in addition to protein-coding transcripts. Performance results were assessed based on a region (~50,000 reads), mouse chromosome 1 (~2.2M reads) and a complete RNA-seq dataset (~30M reads). RNA-eXpress achieved similar performance as Scripture and significantly faster runtime when compared to Cufflinks. Performance calculations were averaged based on three runs on Quad-core Desktop machines (Fedora core 16, 8GB RAM). Accuracy was determined by comparison to human annotated gold standard on region E2 of mouse chromosome 5. Comparisons are recorded as N/A where a program is not capable of performing this analysis.


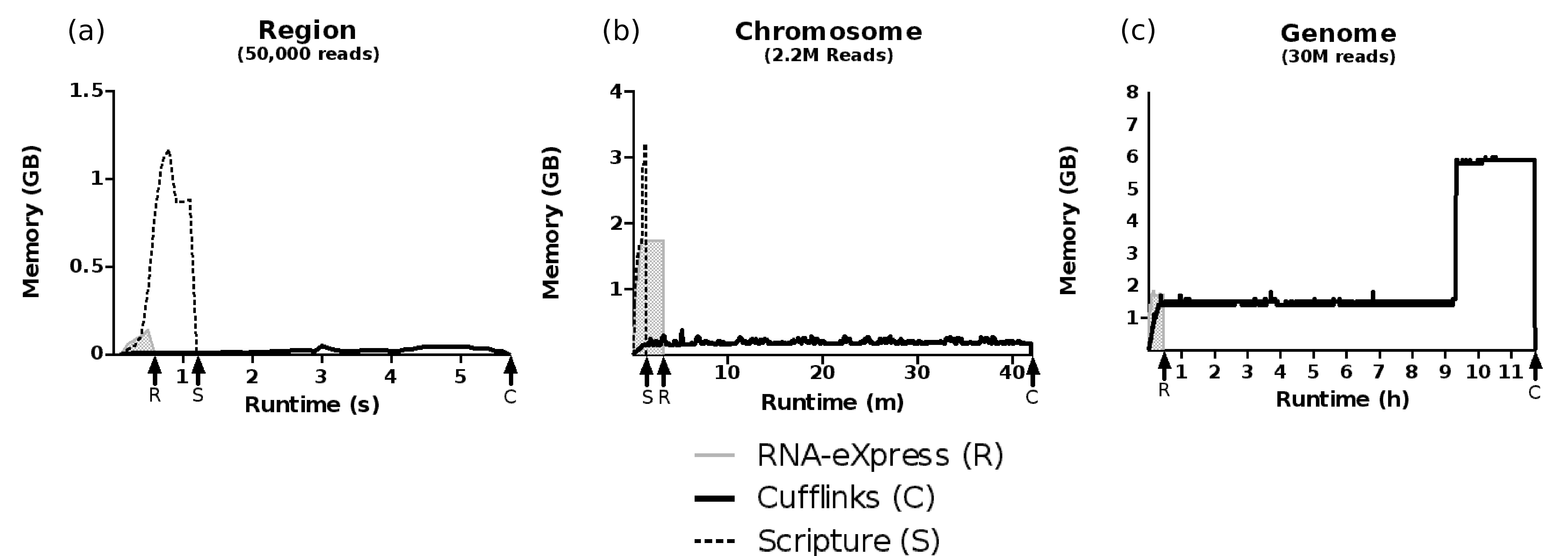


**Supplementary Figure 1a: Memory and Runtime Comparison of RNA eXpress, Cufflinks and Scripture,** demonstrating memory usage and run time on a desktop computer comparison between RNA-eXpress (R), Cufflinks (C) and Scripture (S) with program completion times denoted by arrows. **(a)** The manually annotated region on mouse chromosome 5 E2 used for quality comparisons (~50,000 reads), **(b)** Reads mapping to mouse chromosome 1 (~2,200,000 reads) or **(c)** A complete RNA sequencing sample (~30,000,000 reads) mapping across the entire genome. Scripture provides functionality to run only individual chromosomes and as such could not be tested in the whole genome comparison.


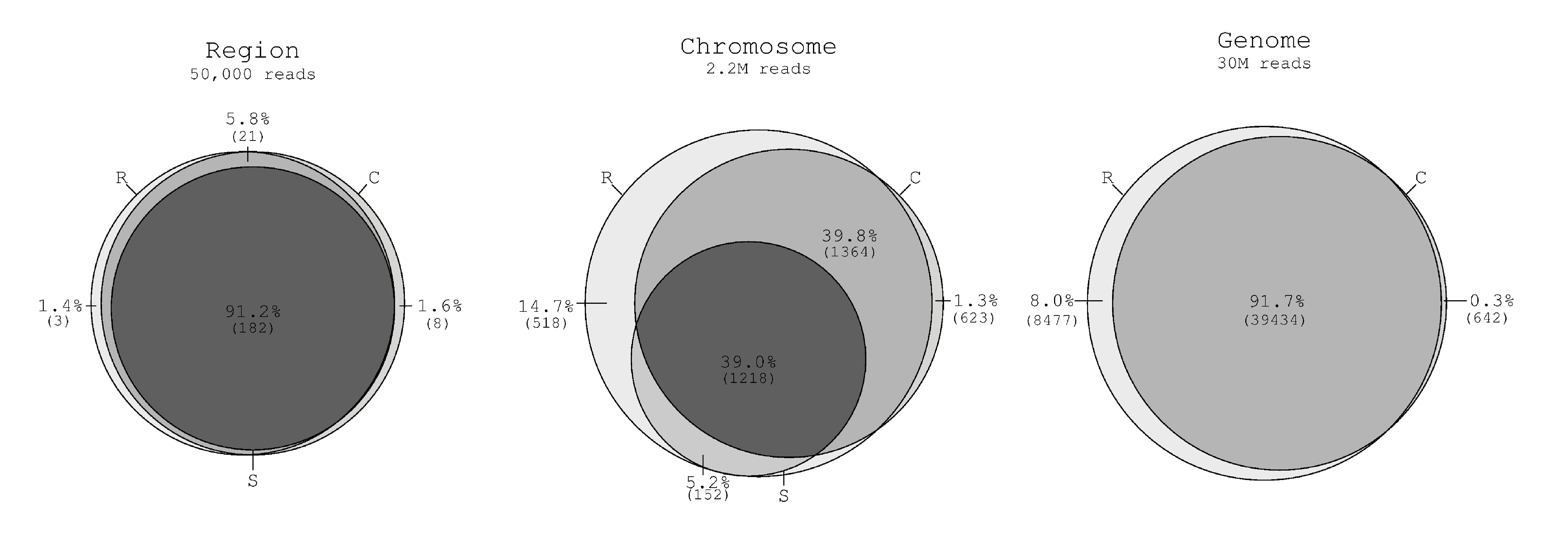


**Supplementary Figure 1b: Feature Identification Comparison between RNA eXpress, Cufflinks and Scripture,** demonstrating identified feature read coverage and feature counts comparing the RNA-eXpress Transcripts algorithm (R), Cufflinks (C) and Scripture (S). Percentages of read counts represent raw number of reads mapping across detected features. The minimum number of predicted features is reported where multiple overlapping features are detected by different algorithms. **(a)** The manually annotated region on mouse chromosome 5 E2 (~50,000 reads), **(b)** Reads mapping to mouse chromosome 1 (~2,200,000 reads) or **(c)** A complete RNA sequencing sample (~30,000,000 reads) mapping across the entire genome. Scripture provides functionality to run only individual chromosomes and as such could not be tested in the whole genome comparison.


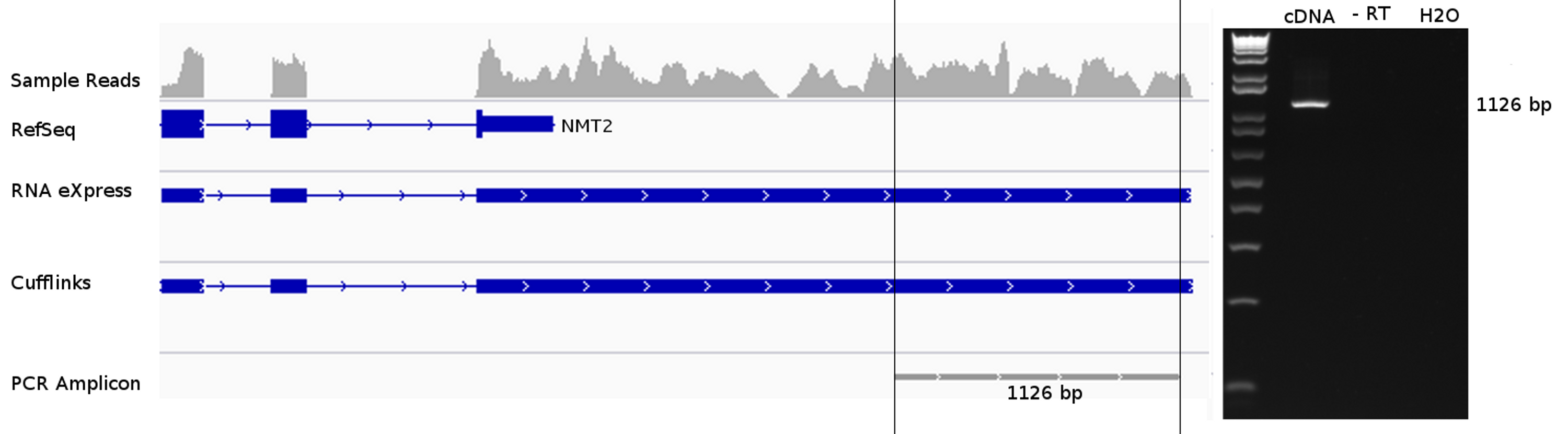


**Supplementary Figure 2a: Nmt2 Transcript Annotation and PCR validation,** illustrating RefSeq, RNA eXpress and Cufflinks gene annotation compare to sequenced sample reads and experimentally validated PCR amplicon. Experimental validation of the 1126bp PCR amplicon demonstrates accurate prediction by both RNA eXpress and Cufflinks of an extended 3’UTR that is not annotated by RefSeq. The amplified PCR band was validated with Sanger based sequencing as containing the sequence predicted by the RNA eXpress program.


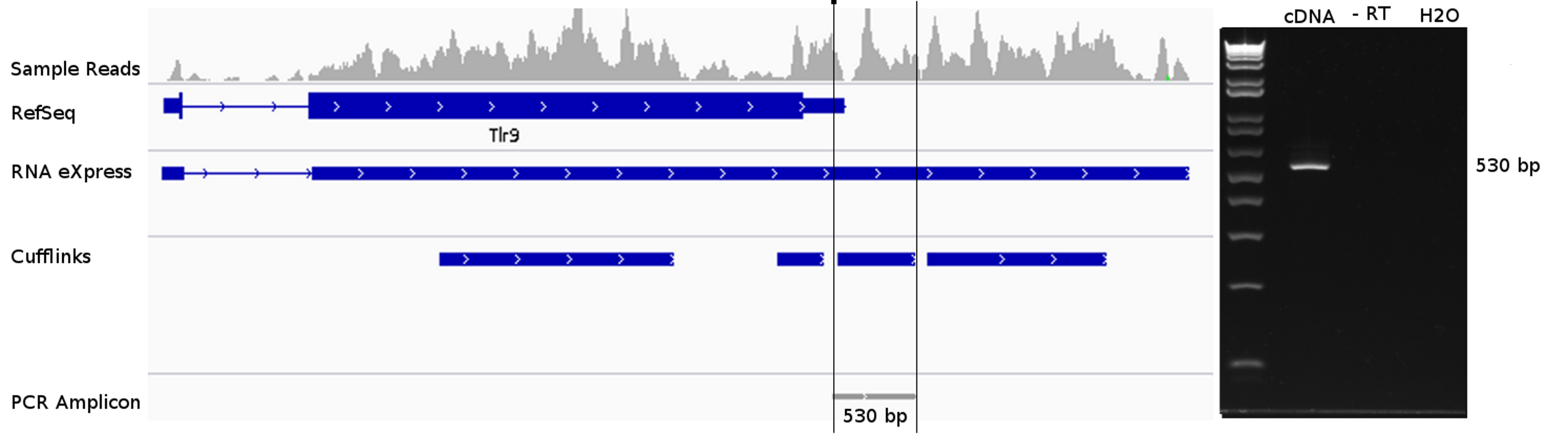


**Supplementary Figure 2b: Tlr9 Transcript Annotation and PCR validation,** illustrating RefSeq, RNA eXpress and Cufflinks gene annotation compared to sequenced sample reads and experimentally validated PCR amplicon. Experimental validation of the 530bp PCR amplicon demonstrates accurate prediction by RNA eXpress of an extended 3’UTR of the gene whereas the multiple short transcripts annotated in the Cufflinks algorithm suggests incorrect annotation of discrete transcriptional units. The amplified PCR band was validated with Sanger based sequencing as containing the transcript predicted by the RNA eXpress program.
